# Notes on Dental Practice

**Published:** 1884-05

**Authors:** 


NOTES ON DENTAL PRACTICE.—By Henry C. Qinby, Licentiate in Dental Surgery of the Royal College of Surgeons in Ireland, etc., etc. With illustrations, Publishers: The S. S. White Dental Manufacturing Co., Philadelphia, 1884, Price, $2.25.

The author, as he states in his preface, has attempted to present in a series of notes his methods as pursued by him in his office practice, leaving the elaboration of theories and the search after causes in the able hands that are now on both sides of the Atlantic pursuing these investigations. He refers to the treatment of the temporary and permanent teeth; extraction as a means of preventing decay; irregularities; amalgam; pivoting; and gutta percha for impressions in eight chapters containing nearly two hundred pages which are lucid and instructive. Dr. Quinby is an advocate for the use of chloroform in dental operations, to the exclusion of what are generally considered in America to be less dangerous anæsthetic agents, although he advises the dentist to avoid such a responsibility as the employment of these agents entail. A great amount of valuable information can be derived from a perusal of this work.

